# HETEROGENOUS AGING OF LOCAL TISSUES IS A MAJOR CONTRIBUTOR TO THE AGE-RELATED DECLINE IN GLYCOSAMINOGLYCANS AND FRACTURE TOUGHNESS OF CORTICAL BONE

**DOI:** 10.21203/rs.3.rs-11379365/v1

**Published:** 2026-10-30

**Authors:** Savannah Heath, Ashley Ridoutt, Michael Reyes, Keying Ye, Jean X. Jiang, Xiaodu Wang

**Affiliations:** 1 Mechanical, Aerospace, and Industrial Engineering, University of Texas at San Antonio, USA; 2 Biomedical Engineering, University of Texas at San Antonio, USA; 3 Management Science and Statistics, University of Texas at San Antonio, USA; 4 Biochemistry and Structural Biology, University of Texas Health Science Center at San Antonio, USA

**Keywords:** Glycosaminoglycans, Bone Quality, Aging, Tissue Aging, Microstructural Heterogeneity, Fracture Toughness, Raman Spectroscopy

## Abstract

Bone fragility fractures increase with age as bone toughness declines, potentially in part due to the loss of glycosaminoglycans (GAGs). However, the mechanisms linking GAG depletion to bone quality remain unclear, partly due to the dynamic and heterogeneous nature of bone remodeling during aging. During bone remodeling, newly formed secondary osteons and older interstitial tissue are randomly distributed throughout the bone, each with distinct material properties. This microstructural heterogeneity adds complexity to studying age-related changes in bone quality that cannot be explained by donor age alone. This study hypothesized that the age-related decline in bulk bone fracture toughness originates from changes in local tissue GAGs with tissue aging and is amplified by an increasing proportion of old tissue relative to new tissue with advancing donor age. To test this, local tissues in human cortical bone samples across different ages and sexes were analyzed using Raman spectroscopy, DMMB, Alcian blue, histomorphometry, microcompression, and fracture toughness testing. The results showed that compositional and mechanical properties of local tissues changed significantly with tissue age, but not with donor age or sex. The volume fraction of old tissue as well as GAG heterogeneity (FWHM) increased significantly with donor age. Furthermore, age-related changes in bulk bone GAG content were strongly correlated with bone fracture toughness. These findings suggest that age-related loss of bone quality arises from the combined effects of tissue age-dependent deterioration in local properties and donor age–dependent accumulation of older tissue.

## 1. INTRODUCTION

Bone fragility fractures, which occur under low-energy loads like falls from standing height, become more common as people age, leading to elevated risks of morbidity, high healthcare costs, reduced quality of life, and even premature death (1–3). While Dual Energy X-ray Absorptiometry (DXA) measured bone mineral density (BMD) is the clinical standard for predicting fracture risk (4), it only accounts for only 50% of variation in fragility fractures (5, 6). In some cases, age-related bone fragility fractures can occur independent of BMD and bone mass loss (7). This discrepancy likely stems from age-related changes in other factors, including the intrinsic properties of local bone tissues.

The age-related increased brittleness and decreased strength in bone, has been extensively correlated with compositional changes in bone matrix. For example, age-related loss of hydroxyapatite (clinically measured *via* BMD) corresponds to reduced bone strength and stiffness (8, 9), while age-related increases in collagen crosslinks, denaturation, and maturity are mainly associated with changes in tissue elasticity and energy dissipation (10, 11). Moreover, decreases in matrix bound water and non-collagenous proteins like proteoglycans (PGs) correspond to reduced bone toughness (10, 12, 13). Located in the extrafibrillar matrix (14–16), PGs like Biglycan and Decorin consist of a core protein linked to negatively charged glycosaminoglycan (GAG) chains (14, 17) that generate osmotic potential to maintain hydration in the bone. Though minor components, PGs and GAGs play critical roles on bone quality by regulating mineralization, collagen assembly, and water retention for plastic energy (10, 12, 13). Unfortunately, a reduction of up to 17% in cortical GAG levels has been observed with aging (18), indicating that they are likely important contributors to the age-dependent decline of bone quality.

While compositional shifts correlate with age-related bone decline, the underlying mechanisms remain unclear, partly because bones are dynamic and constantly remodeled. During human bone remodeling, osteoclasts resorb old bone and osteoblasts refill the gap with new bone, replacing around 5% of cortical bone and 12% trabecular every year (19). In cortical bone, this process forms distinct structures called osteons. Consequently, bone tissue consists of a heterogeneous mix of new (secondary osteons) and old (interstitial) tissue, reflecting diverse tissue ages across locations.

Different regions within a bone are both compositionally (20, 21) and mechanically distinct (22, 23). Additionally, because bone formation is delayed with age (24, 25), older donors have a smaller fraction of new tissue and a higher volume of older tissue. Thus, both delayed tissue formation and ongoing matrix aging alter bone’s overall composition and mechanics, yet the specific impact of local tissue aging on bulk bone quality remains poorly understood.

To this end, we hypothesized that the age-related decline in bulk GAG content and corresponding mechanical behavior originates from changes in local tissues (secondary osteons vs. interstitial tissues) which is amplified by shifts in microstructural proportions of new and old bone. To test this hypothesis, we examined how donor age and sex influence GAGs along with other compositional properties of relative, local bone tissues ages. Then, we examined whether the fraction of older tissue increases with donor age, investigated the relationship between local heterogeneity and bulk GAG content, and evaluated how these local shifts correlate with age-related loss in bulk bone fracture toughness.

## 2. METHODS

### 2.1 Overview:

An *ex vivo* human cadaver bone model was used to test these hypotheses, utilizing diaphyseal femurs from donors in three gender-matched age groups: young (<40 years), middle-aged (40–65 years), and elderly (>65 years) (**Table S1**). Two sets of experiments were implemented of gender-matched age groups. First, cortical bone samples (n=45) underwent three-point bending to determine bulk fracture toughness. Post-fracture, the left side was powdered to assess bulk GAGs via DMMB assay, while the right side was sliced for histomorphometric analysis to quantify microstructural heterogeneity (the fractional ratio of secondary osteons versus interstitial tissues). Moreover, the local compositional properties of secondary osteons (newly formed) and interstitial (older) tissue were evaluated for the same slices using Raman spectroscopy. To capture GAG distribution heterogeneity beyond discrete histomorphometry, a higher-resolution subset analysis was performed on 12 representative samples. Alcian blue-stained bone slices yielded GAG intensity histograms; because these were normally distributed, full width at half maximum (FWHM) was calculated to quantify areal heterogeneity and evaluate tissue variance effects on bulk integrity. Second, bone cores from 53 donors (n=53) were subjected to uniaxial microcompression to assess local mechanical properties of secondary osteons and interstitial tissue.

### 2.2 Three-point bending fracture toughness test:

Longitudinal, rectangular cortical bone strips (2×4×20 ±0.02mm) were cut from the femoral diaphysis (**Fig. S1B**). Then samples were razor-notched and fatigue pre-cracked (2000 cycles, load ratio of 0.1=2/20N). Before testing, samples were kept in 4° overnight in 1×PBS to ensure they were fully hydrated. Since bone exhibits both linear and plastic behavior, resistance curve (*R*-curve) was used to characterize fracture resistance as a function of crack extension. Following ASTM E-1820 -A1, the single edge notched beams were subjected to 3-point bending on a micromechanical tester (Deben Microtester) at 0.033mm/min under displacement control in multiple consecutive cycles to determine their *R*-curves. In each cycle, samples were loaded until the force reached a maximum and then unloaded to 50% of the force. 10-15 loading-unloading cycles were performed per sample. Samples were hydrated throughout the experiment with 1xPBS.

In this study, *J*-integral was used to evaluate the fracture toughness of bone, which was assessed following a cyclic loading scheme with a single specimen approach (**Fig. S1B**) (26). The value of *J_i_* at the *ith* cycle was calculated using the following equations (26).

(1) $$J_{i}=J_{el}+J_{pl}=\frac{K_{i}^{2}\left(1-v^{2}\right)}{E}+\frac{1.9A_{pl}}{B(W-a)}$$

where $J_{el}$ and $J_{pl}$ are the elastic and plastic part of the *J*-integral, respectively. *K_i_* is the stress intensity factor, *ν* is Poisson’s ratio, *E* is elastic modulus, *B , W*, and *a* are the thickness, width, and crack length of the specimen, respectively, and *A_pl_* is the plastic portion of the area under the load-displacement curve for the respective cycle. For simplicity, while preserving reasonable accuracy, the elastic modulus (E) and Poisson’s ratio (ν) of human cortical bone were approximated as 18 GPa and 0.3, respectively (27).

$K_{i}$ at the *ith* loading/unloading cycle was calculated using the following equation (26).

(2) $$K_{i}=\frac{P_{i}S}{BW^{\frac{3}{2}}}f\left(\frac{a_{i}}{W}\right)$$

where *P_i_* is load, *S* is supporting span, *W* is specimen width, *B* is specimen thickness, and *a_i_* is the crack length at the *ith* cycle. Crack length *a_i_* was estimated from the unloading compliance slope ($C_{i}=\Delta \delta_{i}/\Delta P_{i}$) following A1.13, using the following equation.

(3) $$\frac{a}{w}=0.999748-3.9504u+2.9821u^{2}-3.21408u^{3}+51.51564u^{4}-113.031u^{5}$$

where, $u=\frac{1}{{\left[\frac{B_{e}WEC_{i}}{S/4}\right]}^{\frac{1}{2}}+1}\cdot f\left(\frac{a_{i}}{W}\right)$ is calculated using the following equation (26).


(4) $$f\left(\frac{a_{i}}{W}\right)=\frac{3{\left(\frac{a_{i}}{W}\right)}^{\frac{1}{2}}\left[1.99-\left(\frac{a_{i}}{W}\right)\left(1-\left(\frac{a_{i}}{W}\right)\right)\left(2.15-3.93\left(\frac{a_{i}}{W}\right)+2.7{\left(\frac{a_{i}}{W}\right)}^{2}\right)\right]}{2\left(1+2\left(\frac{a_{i}}{W}\right)\right){\left(1-\left(\frac{a_{i}}{W}\right)\right)}^{\frac{3}{2}}}$$

The *J*-integral values obtained from consecutive loading cycles were plotted against crack length (*a*) to generate a resistance curve, *R*-curve (**Fig. S1B**). From the *R*-curve, the initiation fracture toughness (*J_IC_*) was determined following the offset method described in ASTM E-1820, with 0.05mm offset blunting line used instead of the value (0.2mm) recommended by the standard to better account for crack blunting and small specimen geometry for bone (28). The propagation fracture toughness (*J_prop_*) was estimated as the slope of the rising *R*-curve between the exclusion lines following ASTM E-1820 (28). *J_prop_* could not be calculated for 7 samples that had early unstable crack growth.

### 2.3 DMMB Bulk glycosaminoglycan quantification:

Sulfated GAGs were quantified using 1.9-dimethylmethylene blue (DMMB) assay. Following established protocol (29), post-fractures strips (~70mg, 2x4x5mm^3^) were crushed into fine powder in liquid nitrogen. Non-mineralized matrix GAGs were extracted with lysis buffer I (4 M guanidine HCl, 0.05 M Tris, and 0.1 M 6-aminocaproic acid, pH 7.4) containing proteinase inhibitors at 4° for 72 hours. Mineralized matrix GAGs were subsequently extracted by treating the remaining bone powders with lysis buffer II (buffer I supplemented with 0.5 M EDTA tetrasodium salt) at 4° on the orbital shaker for 72 hours. Supernatants were collected, filtered via 3 kDa MWCO centrifuge filters to remove buffer reagents, and mixed (50μL) with 200μL DMMB mix (46 mM DMMB (Sigma-Aldrich), 40 mM NaCl, and 40 mM glycine, and the pH adjusted to 3.0 with acetic acid) in 96-well plates. The absorbance was immediately measured at 525 nm and GAG content was quantified using chondroitin sulfate standard curves.

### 2.4 Bone histomorphometric characterization:

Post-fracture bone slices (**Fig. S1C**) were sequentially lapped and polished using alumina abrasives to a final dimension of 2.0mm×4.0mm in size and 0.15mm in thickness, with a surface roughness within a range of 50-100nm, and images were taken of each slice.

Using ImageJ, total bone slice area as well as Haversian and Volkman pores were measured. Additionally, the pores of the canals were converted into a mask for later subtraction from osteon areas. Next, all osteons were manually traced along the outer cement line and independently added to an overlay in ImageJ’s *ROI manager* where it was assigned a number value. The masks of the canals were then added back to the image containing the osteon overlays. Then, shape descriptors for all osteons, including the pore area fractions, were measured and exported. Tissue type classification was implemented by blinded lab members trained in bone histomorphometry. Finally, different tissue types were classified into the following categories:

#### New secondary osteons:

aligned with the long axis of the bone were identified by their intact cement line (>90%) and a smooth Haversian canal perimeter to calculate the total area fraction of newly formed tissue (‘New Tissue’). Classification of secondary osteons included both circular (aspect ratio <2) or elongated morphologies (aspect ratio >2) (21, 30). Osteons with large Volkmann systems, pore area fraction >25%, or located on the edge of the bone slice were excluded.

#### Old tissues:

the tissue between secondary osteons and classified into two subtypes, *i.e.*, fragmentary osteons and initial interstitial tissues. *Fragmentary osteons* exhibit an incomplete shape of osteons or remnant of previous remodeling, having incomplete cement line or show greater than 10% resorption in the Haversian canal, suggesting that they are actively undergoing the bone resorption process (31). *Interstitial tissues* are deemed as remnants of primary bone after bone remodeling with characteristics of primary bone or little to no consistent patterns. Both subtypes were collectively considered as ‘Old Tissue’ in this study.

#### Bone porosity:

Bone porosity (*P_o_*) was approximated by calculating the ratio of the total Haversian canal area to the total cross-sectional area of the sample.

#### Areal fraction ratio between new and old tissues:

Areal fractions of new and old tissue within each section were calculated first, and then the areal fractional ratio ($R_{N/o}$) between the new tissue ($A_{\mathrm{new}}$) and the old tissue ($A_{\mathrm{old}}$) was calculated as follows:


(5) $$R_{N/o}=A_{\mathrm{new}}/A_{\mathrm{old}}$$

This ratio was assessed with respect to the solid tissue only, excluding Haversian systems (voids) to mitigate the confounding effects of porosity on the analysis. It was used to characterize the average tissue age of the bone, with higher values indicating a relatively younger bone tissue.

### 2.5 *Alcian Blue characterization of local and bulk GAGs* (Fig. S1E):

For a higher resolution characterization of the heterogeneity of GAGs in bone, 12 bone slices from 6 gender-matched young and 6 old samples were stained with Alcian blue to measure GAGs using a protocol reported elsewhere (32). Briefly, the bone samples were demineralized for 48hours in 10% EDTA before fixation with 4% paraformaldehyde for 24 hours. The samples were then washed in 1× PBS then dH_2_O before incubation in 3% acetic acid for 3 min. The prepped bone slices were stained in 1% Alcian blue 8GX (Sigma-Aldrich) for 4hours, and washed with 1 × PBS for three times each for 5 min. After staining, the samples were destained, fixed, and imaged. Using ImageJ, histograms of the GAG intensities were generated. Shapiro Wilk test was first conducted to determine normality of each sample. Then the FWHM was measured. Local GAG intensities of new secondary osteons and old tissue were taken from the average of 50 point-intensities per bone slice.

### 2.6 *Raman Spectroscopy characterization of local GAGs and other compositional properties* (Fig. S1D):

Compositional analyses were performed on the same bone slices using a confocal Raman spectroscopy system (Horiba LabRAM HR Evolution) following established protocols (33). Spectra were acquired using a 785 nm laser focused through a 50x objective (NA = 0.75, ~5μm in spot size). For each slice, three spectra (300–1800cm^−1^, 20s acquisition) were taken across 12 locations: 6 secondary osteons and 6 interstitial tissues, respectively. Spectra for the osteons were taken approximately in the central cortex between the cement line and the haversian canal. Spectra were averaged for each tissue type, background subtracted with a 4^th^ order polynomial, and filtered via 2^nd^ order Savitzky-Golay polynomial (window size: 25pts). Peak intensity measurements were extracted from processed spectra using a custom MATLAB script (33).

In this study, the following Raman properties were measured to characterize compositional changes in bone. These properties included mineral-to-matrix ratios (ν_1_PO_4_/Amide I, ν_1_PO_4_/CH_2_-wag, ν_1_PO_4_/Proline, and ν_2_PO_4_/Amide III), crystallinity (1/FWHM of ν_1_PO_4_ at 960cm^−1^: the inverse of the full line-width of the ν_1_PO_4_ peak at half the maximum height relative to zero intensity), organic helical status (1665/1635cm^−1^), organic matrix maturity (1665/1690cm^−1^), glycosaminoglycan content (GAGs/PO_4_ and GAGs/Amide I), and post-translational modifications to the organic phase (Hydroxyproline/Proline). With these measures, the quantity and quality of the mineral and collagen phases could be evaluated. For better visual comparisons only (Fig. 1), each Raman measurement was normalized (X-min)/(max-min).

### 2.7 *Microcompression tests of local mechanical behavior* (Fig. S1F):

As described previously (34), for the microcompression tests, 1.5mm thick cross-sections cut from femoral diaphysis were glued with cyanoacrylate (BSI Adhesives Insta-Cure +) onto a bovine bone support during the coring process. Cylindrical microcores (200μm in diameter, 500μm in length) of interstitial tissue and longitudinal secondary osteons were prepared using a benchtop CNC milling machine (Light Machines proLIGHT) equipped with a microscope attachment. Regions of interest were identified under microscopic guidance and centered within a preset ring matching the coring bit diameter and CNC coordinates. Coring was performed at a feed rate of 10μm per revolution with a spindle speed of 1000 rev/min under continuous irrigation with PBS. Two interstitial and osteonal microcores were each obtained per donor.

Bone cores underwent uniaxial compression testing following established methods. (34). Specimens were placed in a custom fixture mounted on a benchtop mechanical testing system (EnduraTEC Elf 3300). Maintained under hydration via a surface-tension water bead, cores were compressed to failure in displacement control at a strain rate of approximately 1% per second, with force and displacement sampled at 5 Hz. Throughout loading, images were captured at 1 frame/s using a stereo-zoom microscope with an attached CCD camera (Motic 3000), and tests were terminated when specimens reached 50% of its original length.

Local strain was extracted from recorded video using custom image processing software, synchronized with applied stress, and smoothed via a rolling average method to generate stress-strain curves. From the stress-strain curve, the elastic modulus (*E*), yield stress and strain $\left(\sigma_{y},\varepsilon_{y}\right)$, ultimate stress and strain $\left(\sigma_{u},\varepsilon_{u}\right)$, failure stress and strain $\left(\sigma_{f},\varepsilon_{f}\right)$, elastic $\left(U_{e}\right)$, post-yield $\left(U_{p}\right)$, and total failure energy density $\left(U_{t}\right)$ were determined. The yield point was determined using the 0.2% offset method of the initial linear portion of the stress-strain curve.

### 2.8 Statistical Analysis:

To investigate the effects of donor age, tissue type, and sex on local bone compositional and mechanical properties, MANOVA tests utilizing Pillai’s Trace were conducted, as Levene’s test indicated that certain measurements (*e.g.*, ν_1_PO_4_/Amide I, carbonate content, crystallinity, and matrix maturity) violated the assumption of homogeneity of variances. Multiple comparisons between test groups utilized the Bonferroni correction method with familywise error rate control. Similarly, MANOVA and post hoc Bonferroni tests assessed the effects of donor age and sex on the areal fractional ratio between new and old tissues $\left(R_{N/O}\right)$, GAGs, and the bulk fracture toughness of bone.

To relate local GAG levels to bulk GAG content and validate new and old tissue classifications, two rule-of-mixture models were employed. Both models quantified local GAG levels using Alcian intensities, with the first model incorporating histomorphometrically derived tissue areas and the second utilizing Alcian distribution FWHM to assess sample heterogeneity. Pearson correlation analyses were conducted for both models and their individual components to determine correlations between true bulk GAG content and local/calculated GAG values.


(5) $$\mathrm{Bulk GAGs/Material}={\mathrm{GAGs}}_{\mathrm{new}}A_{\mathrm{new}}+{\mathrm{GAGs}}_{\mathrm{old}}A_{\mathrm{old}}={\mathrm{GAGs}}_{\mathrm{old}}+\mathrm{FWHM}$$

Lastly, general linear models assessed the relationships between the bulk fracture toughness properties (J_IC_ and J_PROP_) and bulk GAGs. Because sex was not significantly associated with crack initiation toughness, it was not included as a covariate for further analysis. Furthermore, since age emerged as a significant covariate for both GAG content and fracture toughness, partial Pearson correlations controlling for age were conducted to examine the direct effects of GAGs on fracture properties.

## 3. RESULTS

### 3.1 GAGs and other compositional properties are primarily dependent on local tissue type, with selective effects of donor age and sex:

The compositional properties of osteons and interstitial tissues (mean ± standard deviation) obtained using Raman spectroscopy are presented in **Table S2** for bone samples from different age and sex matched groups.

Multivariate Analysis of Variance (MANOVA) revealed that tissue type had a significant effect on all compositional properties except Hyp/Pro (Table 1). In contrast, donor age influenced only a few compositional properties, including mineral quantity (PO_4_/CH_2_, PO_4_/Amide I), mineral quality (CO_3_ content), and organic phase quality (GAGs/PO_4_). The effect of sex was even more restricted to organic phase properties (GAGs/PO_4_, GAGs/Amide I, and Helical Status). No interaction effects among donor age, sex and tissue type on compositional properties were observed.

The *post hoc* comparisons (Bonferroni) revealed that interstitial tissues had higher mineral- -to-matrix ratios (PO_4_/CH_2_, PO_4_/Amide I, PO_4_/Pro, PO_4_/Amide III) compared to secondary osteons (*p* < 0.001) (Fig. 1). Furthermore, the quality of the mineral phase was also significantly different between these two tissue types, showing a significant decrease in CO_3_ content (*p* < 0.05) and a significant increase in crystallinity (*p* < 0.05). Similarly, significant differences in the organic phase were observed between the two tissue types, showing higher GAGs/PO_4_ and GAGs/Amide I (*p* < 0.05), but lower helical status secondary osteons compared to interstitial tissues (*p* < 0.05). However, no significant difference in Hyp/Pro was observed between the two tissue types (*p* > 0.05). These results indicated that tissue age had significant effects on the quantity and quality of both mineral and organic phases in local bone tissues.

The effects of donor age and sex on the compositional properties of local tissues were also observed. Young bone had higher mineral-to-matrix ratios (PO_4_/CH_2_, PO_4_/Amide I) and lower carbonate content (CO_3_ content), compared to bone samples from the middle-aged and old donors (Table 1). In addition, young bones had lower GAGs/PO_4_ compared to middle-aged bone only. Lastly, males had higher values of GAGs/PO_4_, GAGs/Amide I, and helical status compared to females.

### 3.2 Mechanical properties of local tissues are dependent only on tissue types:

The results of the microcompression tests are summarized in Table 1. MANOVA analyses revealed that among donor age, sex, and tissue type, only tissue type (secondary osteons vs. interstitial tissue) had a significant effect on $\varepsilon_{f},Up$, and *U_t_*, parameters that primarily reflect the ductility and toughness of bone tissue. In addition, significant interaction effects were found between sex and donor age on plastic energy dissipation (*U_p_*), and between donor age and tissue type on ultimate strain $\left(\varepsilon_{u}\right)$. *Post hoc* Bonferroni comparisons showed that secondary osteons, which represent newly formed bone tissue, exhibited significantly higher plastic energy dissipation (*U_p_*), toughness (*U_t_*), and strain at failure $\left(\varepsilon_{f}\right)$ compared to interstitial tissue (*p* < 0.05). These findings suggest that older tissue is more fragile than newly formed tissue in bone.

### 3.3 Heterogeneity of local GAGs in bone are affected by age and sex:

MANOVA analyses indicated that donor age had significant effects on bulk GAGs as well as heterogeneity assessed via areal fractions and FWHM (*p* ≤ 0.05) (Table 2). On the other hand, sex appeared to have no significant effect on these measurements (*p* > 0.05). In addition, an interaction between age and sex was observed only for the tissue age ratio ($R_{N/O}$) (*p* < 0.03).

Moreover, *post-hoc* analysis (Bonferroni) revealed that GAGs as well as the areal fraction of new tissue (total secondary osteons) decreased with donor age (Fig. 2). As a result, the areal fractional ratio ($R_{N/O}$) between new to old tissue decreased with donor age, suggesting an elevated average age of local tissue as people age. In addition, the porosity was found to increase with donor age, consistent with previous reports (35, 36).

### 3.4 Local matrix GAGs are associated with bulk GAGs content through rule of mixture:

The distribution of GAGs with aging is shown in **Figure S1E**. Visually, in agreement with the Raman spectroscopy data, the new osteon locations had the highest GAG content and were very similar for all osteons regardless of age. The lowest values corresponded with the interstitial tissues, although the minimum value decreased with age. MANOVA revealed (Table 2) that the mean GAG intensity, reflecting the bulk GAG content, decreased with age, while the FWHM of the distribution increased with age. Sex did not have a significant effect on GAGs, although males had a marginally higher bulk GAG content (p-value =0.07) similar to the Raman spectroscopy results.

Pearson correlations (Table 3) demonstrated that both rule of mixture models were significantly associated with the measured Alcian GAG intensity regardless of whether tissue heterogeneity was quantified using histomorphometrically measured areas or the histogram FWHM. Further correlations into discrete properties reveal that GAG levels for osteons and interstitial tissues were both significantly associated with bulk GAGs, although the association was much stronger for older, interstitial tissues. Moreover, the heterogeneity (FWHM) showed the strongest association with bulk GAGs (R^2^=0.77, p<0.001) compared to individual osteon or interstitial components.

### 3.5 Both age and sex significantly affect the bulk fracture toughness of bone:

The descriptive statistics and the effects of age and sex on the bulk fracture toughness of bone are shown in **Table S3 and** Table 4 respectively. MANOVA analyses indicated that both initiation (*J_IC_*) and propagation (*J_prop_*) fracture toughness of bone changed with donor age, while sex exhibited a significant effect only on propagation fracture toughness (*J_prop_*). In addition, no interaction effects on bulk fracture toughness of bone were observed between donor age and sex.

*Post hoc* multiple comparisons (Bonferroni) showed that both the crack initiation (*J_IC_*) and crack propagation (*J_prop_*) fracture toughness of old bone were significantly lower compared to young bone (*p* < 0.05) (Fig. 2B). In addition, such differences appeared to be greater in crack propagation fracture toughness, *J_prop_* (20–27%) than in crack initiation fracture toughness, *J_IC_* (13–16%) (Table 2).

### 3.6 Bulk GAGs are associated with fracture toughness:

As shown in Table 5, DMMB bulk GAGs exhibited the strongest positive correlations with *J_IC_*. Moreover, after controlling for age, bulk DMMB GAGs remained positively correlated with *J_IC_*. Additionally, the areal fraction of new tissue (intact osteons) was positively correlated with crack initiation toughness, while porosity was negatively correlated. The tissue age ratio exhibited the highest correlation among the areal fraction measures; however, these associations disappeared when controlling for age. Aside from age, *J_PROP_* was not significantly associated with any other explanatory variables.

## 4. DISCUSSION

It is widely accepted that the age-related increase in bone fragility results from two major factors: bone mass loss and the deterioration of bone tissue quality (4, 8, 37). While remodeling imbalance accounts for bone mass loss (8), local tissue heterogeneity complicates bone quality changes (19). This study reveals that local bone properties depend primarily on tissue age rather than donor age or sex; older interstitial tissue exhibits altered matrix composition and reduced mechanical competence compared to secondary osteons. Furthermore, the results of this study suggest that the fraction of older, GAG-poor tissue increases with donor age, resulting in a greater proportion of local bone tissue with inferior mechanical properties, consequently leading to a deterioration of the overall mechanical competence of bulk bone tissue.

The results of this study demonstrate that tissue age-related changes in local bone tissues are manifested in both compositional and mechanical properties. Microcompression tests revealed significant decreases in failure strain, plastic energy, and toughness in old interstitial tissues compared with new secondary osteons. These findings align with *in vivo* animal fluorochrome models where tissue age correlates with increased mineralization, crystallinity, and collagen crosslinks, alongside decreased carbonate and non-collagenous proteins (38, 39). However, unlike tissue age, donor age exhibited very limited influence on local mechanical properties (Table 2). This indicates that the deterioration of local tissue toughness and ductility is driven primarily by tissue aging independently of donor age.

Notably, no significant differences in elastic properties were observed between secondary osteons and interstitial tissue, contrasting with previous micro- and nanoindentation findings (22, 23, 40). This discrepancy likely stems from testing length scales: micro- and nanoindentation assess sub-lamellar behavior, whereas our microcompression tests evaluate entire osteons and interstitial regions. Consequently, sub-lamellar spatial variations in elasticity (23, 40, 41) may have a limited impact on the overall elastic properties measured at the osteonal or interstitial scale.

Compositionally, old interstitial tissue exhibited higher mineral-to-matrix ratios, crystallinity, helical structure, and matrix maturity, alongside lower carbonate and GAG levels (GAG/PO_4_ and GAG/Amide I), highlighting the influence of tissue age (Table 2 **& Fig. S1**). These shifts reflect alterations in both mineral and organic phases, with mineral changes likely driven by rapid deposition over carbonate substitution (8, 20, 42) and collagen modifications stemming from denaturation, non-enzymatic crosslinking, or post-translational modifications (42, 43). Interestingly, our results found decreases in GAGs/PO_4_ and GAGs/Amide I ratios in older (interstitial) tissues, consistent with the Alcian blue results. This suggests that GAG loss is associated with increasing tissue age, although the underlying mechanisms remain to be elucidated in future studies. Intriguingly, GAG loss was more pronounced in the GAG/PO_4_ ratio than in the GAG/Amide I ratio, suggesting that GAG depletion is more closely associated with the mineral phase than with the organic phase in bone. Because GAGs in the mineral phase play a pivotal role in local bone tissue toughness (13, 44), tissue aging–induced GAG loss from the mineral matrix likely represents a key mechanism driving the age-related deterioration of bone fragility.

In contrast, donor age influenced only specific parameters related to mineral content (PO_4_/CH_2_, PO_4_/Amide I), carbonate content, and the glycosaminoglycan-to-mineral ratio (GAG/PO_4_), independently of tissue type (Table 2). The mineral-to-matrix ratio (PO_4_/Amide I) decreased in local tissue with donor age (**Fig. S1**), likely because newly formed osteoid in older individuals contains fewer osteocytes essential for local mineralization (24, 25). Additionally, males exhibited slightly greater differences in organic quality measures (helical structure, GAGs/Amide I, and GAGs/PO_4_ ratios), aligning with previous reports (34, 37), though warranting further research to fully characterize sex-dependent organic changes.

Although bulk bone toughness decreases with age, local tissue quality loss only degrades bulk bone competence if the underlying tissue age distribution shifts. Our findings show that average tissue age increases with donor age, as indicated by a reduced new-to-old tissue ratio ($R_{N/O}$) and elevated Alcian FWHM, aligning with literature on declining bone-turnover efficiency. While some studies report no age-related change in total osteon counts (45) and others observe a decline in newly formed tissue area (36, 46), these apparent discrepancies reflect methodological variations in measuring osteonal volume. Specifically, while total osteon population density can increase with age, the relative area occupied by new tissue simultaneously declines (47, 48), and our study reconciles these differences by calculating areal fractions exclusively with respect to solid tissue while excluding all pores. Furthermore, both FWHM and area-based classifications robustly predicted bulk mean GAGs, with FWHM showing the strongest association. Thus, the decline in bulk bone quality likely originates from increased mean tissue age, leading to a greater proportion of local tissue with inferior mechanical competence compared to newly formed bone.

Finally, bulk tissue GAGs and $R_{N/O}$ were both positively associated with crack initiation toughness (*J_IC_*), indicating that greater proportions of mechanically superior, high-GAG new tissue enhance fracture resistance. While porosity showed a weak correlation with fracture toughness in our histomorphometry data (contrasting with some micro-CT studies due to methodological variations) (2, 36, 49), our results demonstrate that mean tissue age plays a pivotal role in bone quality independently of porosity. Importantly, current anti-resorptive therapies, the gold standard for treating age-related fragility fractures, do not address the detrimental effects of tissue aging on fracture risk. These findings highlight tissue aging as a promising target for therapeutic strategies aimed at preserving bone quality.

This study has some limitations. First, Raman spectroscopy provides relative compositional comparisons via peak normalization rather than absolute quantification and exhibits lower sensitivity to low-concentration molecules. Alcian blue and DMMB were used as complementary methods to further assess GAGs, although it should be noted that both methods require substantial processing of the bone tissue Second, using two partially matched donor groups limited the matched sample size (n = 21) and statistical power for cross-correlating local mechanical properties with compositional metrics and bulk fracture toughness. Third, because cortical cross-sections were optimized for tissue-type separation and focused on transverse fracture toughness and longitudinal compression, the anisotropic nature of bone warrants future investigation across other loading modes and orientations. Finally, while tissue age showed the strongest association with tissue properties, further studies are required to elucidate the specific physical, biochemical, or cell-mediated mechanisms driving tissue aging.

## 5. CONCLUSION

Overall, our results show that GAG content as well as other compositional and mechanical properties of local bone tissue change significantly with tissue age, with more limited effects of donor age or sex. The mean tissue age of cortical bone increases markedly with donor age, as evidenced by a decreasing new/old tissue ratio and FWHM of Alcian Blue-stained GAGs. Moreover, bulk GAG content is strongly correlated with the crack initiation toughness of bone. Taken together, these findings suggest that a predominant pathway contributing to age-related loss of bone toughness may originate from an increased fraction of old tissue, whose compositional and mechanical properties are compromised by tissue aging. This deterioration in local tissue quality may ultimately leads to reduced bulk bone toughness.

## Supplementary Material

This is a list of supplementary files associated with this preprint. Click to download.

- SupplementalInformation.pdf
- GraphicalAbstract.png

**Figure S1**. Schematics of experimental setup: **(A)** The diaphysis of human femurs was sectioned into 20 x 2 x 4 mm^3^ rectangular strips aligned with the longitudinal axis for experiments described in B-D, and a 5-mm-thick cross-section slices were dissected for the experiment described in E. **(B)** Three-point fracture toughness tests were conducted on single edge notched specimens in a Deben micromechanical tester to determine R-curves by measuring the J-integral using the single sample method and estimating crack length using the unloading compliance method recommended in ASTM E-1820-A1. **(C)** Bone histomorphometry was used to classify tissue types in the slices from post-fractured specimens. **(D)** Raman spectroscopy was performed to characterize the compositional properties of secondary osteons and interstitial tissue using bone slices obtained from specimens after fracture toughness testing. **(E)** Alcian blue-stained bone sections from young and old male female donors, with histograms averaged from 6 sections from young and old donors. **(F)** Microcompression tests were performed to determine mechanical properties of bone cores from secondary osteons and interstitial tissues extracted from the cross-sectional slices.

**Figure S2.** Raman spectroscopy. **(A)** Secondary osteons and interstitial tissues for human cortical bone. **(B)** Processing method using peak intensity and integrated area. Raw spectra for each group were averaged (black) and fitted with a 4^th^-order polynomial curve to subtract background fluorescence (BG). Then a Savitzky-Golay (S-G) filter (2nd order, window size 25) was applied (blue) or not applied (red). **(C)** Peak ratios were calculated by dividing the highest peak intensity (PI) by another PI.

**Figure S3**. Determination of tissue types and their fractions in bone. (**A**) Original image of polished bone slices, (**B**) ImageJ output of classified osteon types (light cream: circular secondary osteons; brown: elongated/atypical osteons, dark brown: fragmentary osteons, and yellow: edge osteons). , (**C**) Post-classified image of osteon types (white: new osteons; dark gray: fragmentary osteons and interstitial tissue, and light gray: edge osteons). (**D**) Formulae for determining tissue type fractions.

## Figures and Tables

**Figure 1.**
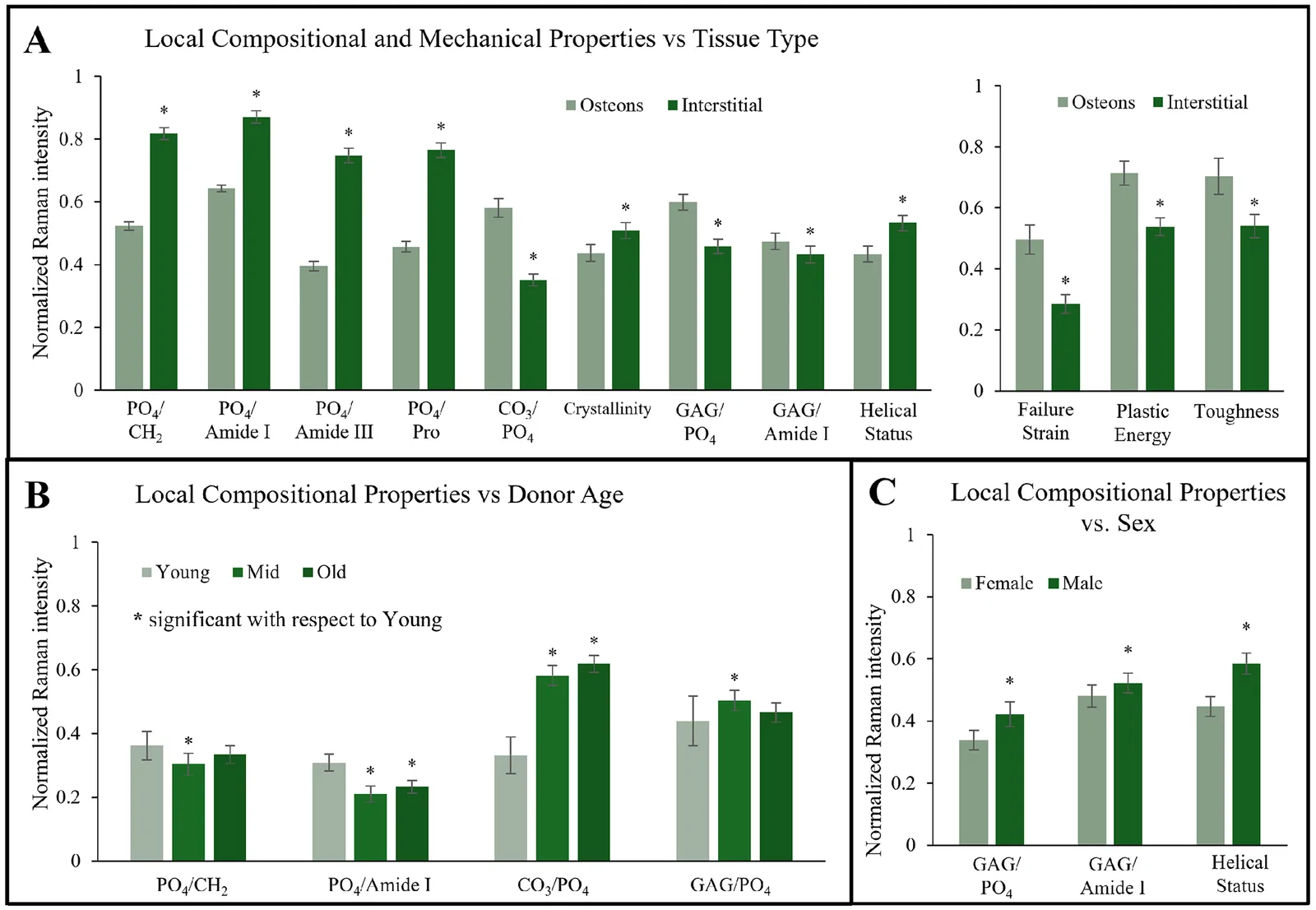
Post-hoc (Bonferroni) comparisons of compositional and mechanical properties of local tissues with (**A**) tissue type, (**B**) donor age, and (**C**) sex. Values for each variable were normalized for visual comparisons between test groups using the following equation: $X_{i}=\frac{x_{i}}{x_{\max}-x_{\min}}$.

**Figure 2.**
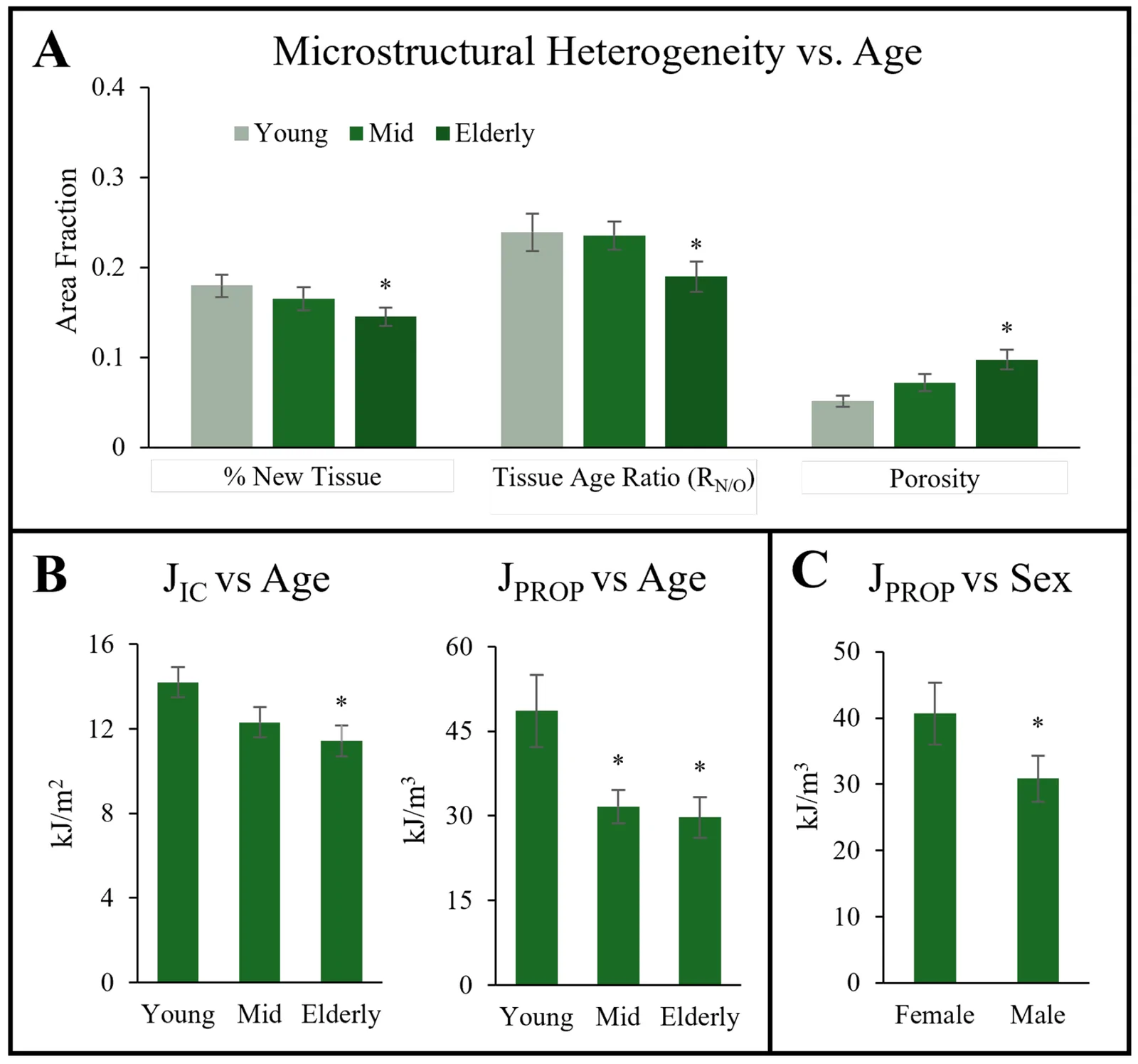
*Post-hoc* (Bonferroni) comparisons of (**A**) microstructural heterogeneity vs. age; (**B**) fracture toughness vs. age; and (**C**) fracture toughness vs. Sex. * Significant with respect to young bone.

**Table 1.** MANOVA results showing the effects of tissue type, age, and sex on the local mechanical and compositional properties of bone (*p*-values). Statistically significant effects are indicated by bold and shaded values (*p* ≤ 0.05).

		Property	Tissue Type	Donor Age	Sex	Sex*Tissue Type	Sex*Donor Age	Donor Age*Tissue Type
**Local Compositional Properties**	Glycosaminoglycans	GAG/PO_4_	**0.004**	**0.01**	**0.034**	NS	NS	NS
		GAG/Amide I	**0.025**	NS	**0.025**	NS	NS	NS
	Mineral Quantity (Mineral/Matrix)	PO_4_/CH_2_	**<0.001**	**0.024**	NS	NS	NS	NS
		PO_4_/Amide I	**<0.001**	**0.005**	NS	NS	NS	NS
		PO_4_/Amide III	**<0.001**	NS	NS	NS	NS	NS
		PO_4_/Pro	**<0.001**	NS	NS	NS	NS	NS
	Mineral Quality	CO_3_ /PO_4_	**<0.001**	**<0.001**	NS	NS	NS	NS
		Crystallinity (1/FWHM PO_4_)	**0.006**	NS	NS	NS	NS	NS
	Organic Quality	Helical Status (1665/1635)	**0.004**	NS	**0.004**	NS	NS	NS
		Matrix Maturity (1665/1690)	**0.02**	NS	NS	NS	NS	NS
		Hyp/Pro	NS	NS	NS	NS	NS	NS
**Local Mechanical Behavior**		Yield Stress (σ_y_)	NS	NS	NS	NS	NS	NS
		Yield Strain (ε_y_)	NS	NS	NS	NS	NS	NS
		Elastic Modulus (E)	NS	NS	NS	NS	NS	NS
		Failure Stress (σ_f_)	NS	NS	NS	NS	NS	NS
		Failure Strain (ε_f_)	**0.013**	NS	NS	NS	NS	NS
		Elastic Energy (U_e_)	NS	NS	NS	NS	NS	NS
		Plastic Energy (U_p_)	**0.012**	NS	NS	NS	**0.047**	NS
		Toughness (U_t_)	**0.039**	NS	NS	NS	NS	NS
		Ultimate Stress (σ_u_)	NS	NS	NS	NS	NS	NS
		Ultimate Strain (ε_u_)	NS	NS	NS	NS	NS	**0.045**

**Table 2.** Effects of age and sex on the distribution of tissue types and GAGs in bone cross-section (mean ± standard deviation), with MANOVA results showing significant effects with bolded values (*p* ≤ 0.05)

MANOVA *p*-values	Age	Sex	Age*Sex
% New Tissue	**0.05**	NS	**0.022**
Tissue Age Ratio **(*R_N/O_*)**	**0.043**	NS	NS
Porosity	**0.004**	NS	NS
Bulk GAG content (DMMB)	**0.041**	NS	NS
Bulk material GAG intensity (Alcian)	**0.022**	NS	NS

**Table 3.** Pearson Correlation (R-values) between local and bulk GAGs in bone, showing statistically significant correlation with bolded values (α ≤ 0.05).

	GAGs_Ost_	GAGs_Int_	FWHM	FWHM + GAGs_Int_	%Ost*GAGs_Ost_ + %Int*GAGs_Int_
Bulk GAGs (Mean Alcian)	**0.627**	**0.840**	**−0.879**	**−0.866**	**0.869**

**Table 4.** Effect of age and sex on the bulk fracture toughness of bone (*p*-values of MANOVA). Statistically significanteffect is with bold and shaded values (*p* ≤ 0.05)

MANOVA p-values	Age	Sex	Age*Sex
**J_IC_**	**0.023**	NS	NS
**J_PROP_**	**0.006**	**0.009**	NS

**Table 5.** Pearson Correlation (R-values) between age, bulk tissue heterogeneity, GAGs, and fracture toughness, showing statistically significant correlation with bolded values (α ≤ 0.05).

R-values		Age	Areal Fractions			DMMB GAGs
			New Tissue Volume	Pores	Tissue Age Ratio (*R*_*N/O*_)	
Pearson Correlation	*J_IC_*	**−0.424**	**0.319**	**−0.301**	**0.345**	**0.56**
	*J_PROP_*	**−0.475**	*0.31*	−0.209	0.26	*0.296*
Partial Pearson Correlation	*J_IC_*	*Control Variable*	0.094	−0.149	0.087	**0.483**
	*J_PROP_*		0.073	0.101	0.116	0.142
